# Two-photon fiberscope with a proactive optoelectrical commutator for rotational resistance–free imaging in freely behaving rodents

**DOI:** 10.1117/1.NPh.12.2.025016

**Published:** 2025-06-18

**Authors:** Yuehan Liu, Jing Zhang, Dian Guo, Yipeng Li, Haolin Zhang, Hyeon-Cheol Park, Wenhe Jing, Hui Lu, Xingde Li

**Affiliations:** a Johns Hopkins University, Department of Electrical and Computer Engineering, Baltimore, Maryland, United States; b Johns Hopkins University, Department of Biomedical Engineering, Baltimore, Maryland, United States; c The George Washington University, School of Medicine and Health Sciences, Department of Pharmacology and Physiology, Washington, DC, United States

**Keywords:** freely behaving rodents, two-photon fiberscopes, neuroimaging, rotational restriction

## Abstract

**Significance:**

The recently developed two-photon (2P) fiberscope offers attractive opportunities in neuroscience by enabling high-resolution neural imaging in freely behaving rodents. However, like other miniature 2P devices, it involves a tether (for fiber and scanner drive wires), which inevitably limits the animal’s movement, especially its rotation.

**Aim:**

We aim to develop a platform for 2P fiberscopes (and other tethered miniature devices), enabling rotational resistance–free neuroimaging in freely rotating/walking rodents.

**Approach:**

We introduced a proactive optoelectrical commutator (pOEC) capable of real-time sensing and compensation for a tiny torque buildup in the tether (with a preselected threshold), preemptively eliminating the rotational resistance when the mouse physically rotates the fiberscope.

**Results:**

Experimental results demonstrated that the pOEC effectively compensates for torque buildup in the fiberscope, thereby maintaining stable 2P imaging performance. In addition, the system minimizes the rotational resistance imposed by the head-mounted tether, enabling near-zero rotational burden during 2P neural imaging in freely behaving mice. Investigations of neural activity further revealed that a considerable proportion of motor cortex neurons exhibited statistically significant changes in their firing patterns when the mouse was restricted by tether-induced rotational resistance or completely immobilized via head fixation.

**Conclusions:**

The results indicated that rotational restriction induced visible impacts on neuronal activities. Our platform offers a promising opportunity for studying dynamic neural circuit functions under nearly natural conditions with minimized impacts by the rotational restriction.

## 1 Introduction

Since the inception of two-photon (2P) fluorescence microscopy in 1990,^1^ it has been broadly used in biomedical fields, notably in neuroscience. With the development of genetically encoded calcium indicators, especially the family of GCaMP,^2^^,^^3^ benchtop 2P microscopy has become a standard technique for visualizing neural activity and structural dynamics in the brains of animals with head restrictions at synapse resolution.^4^^,^^5^ Although promising, head-restrained animal models^6^^,^^7^ are limited in studying normal behaviors such as locomotor gaits, spatial navigation, or social interactions.

The past decade has witnessed significant progress in the development of miniature 2P imaging devices.^8^ The use of miniature objectives or gradient-index lenses has substantially reduced the size and weight of microscopes.^9^ In addition, a variety of fiber-scanning technologies have been demonstrated for recording fine neuronal structures.^10^^–^^12^ With the introduction of a second collection fiber^13^^–^^16^ or a customized double-clad fiber (DCF),^17^^–^^20^ fully integrated flexible 2P fiberscopy has been achieved. The ultracompact size and light weight enable fiberscopes to be head-mountable and detachable, offering opportunities for repeatable functional neuroimaging in freely behaving animals.^15^^,^^21^^–^^23^ Furthermore, fiberscopes eliminate the aberrant sensory feedback caused by head fixation, such as stress and fatigue, allowing functional neural imaging under nearly natural physiological conditions. To the best of our knowledge, all existing miniature 2P imaging devices involve a tether (for the optical fiber and/or the beam scanner drive wires), which can restrict the animal’s movement, particularly its rotation, and may be prone to twist-breaking during imaging.

To overcome these limitations, we have developed a novel proactive optoelectrical commutator (pOEC) for 2P fiberscope, enabling neuroimaging in freely rotating/walking mice with almost no rotational resistance to the animal. By integrating a sensitive torque sensor and a real-time feedback control, the pOEC can proactively sense and compensate for a tiny torque built up in the fiberscope (with a preselected threshold) by rotating the fiberscope along the same direction with a corresponding tiny angle. This effectively nullifies the torque accumulated in the fiberscope, thereby maintaining stable 2P imaging quality and freeing the mouse from rotational constraints. The performance of the pOEC has been assessed by analyzing the movement of mice, and the results demonstrated that the pOEC-based fiberscope generates nearly zero rotational resistance for 2P neuroimaging in freely behaving mice. The impact of rotational resistance on motor cortex neurons was investigated by performing neuroimaging in freely behaving mice (with torque compensation enabled or disabled) and head-fixed (but freely walking) mice. The results indicated a large population of neurons was affected by rotational resistance, and the proportion of affected neurons increased with higher rotational resistance (from torque compensation enabled to disabled to head-fixed). We anticipated the pOEC-based platform provides nearly native “free-behaving” conditions (e.g., with zero rotational resistance to the animal) for functional neuroimaging and benefits a wide range of brain dynamics research, such as spatial navigation and social interaction.

## 2 Methods

### 2.1 pOEC System

**Fig. 1:**
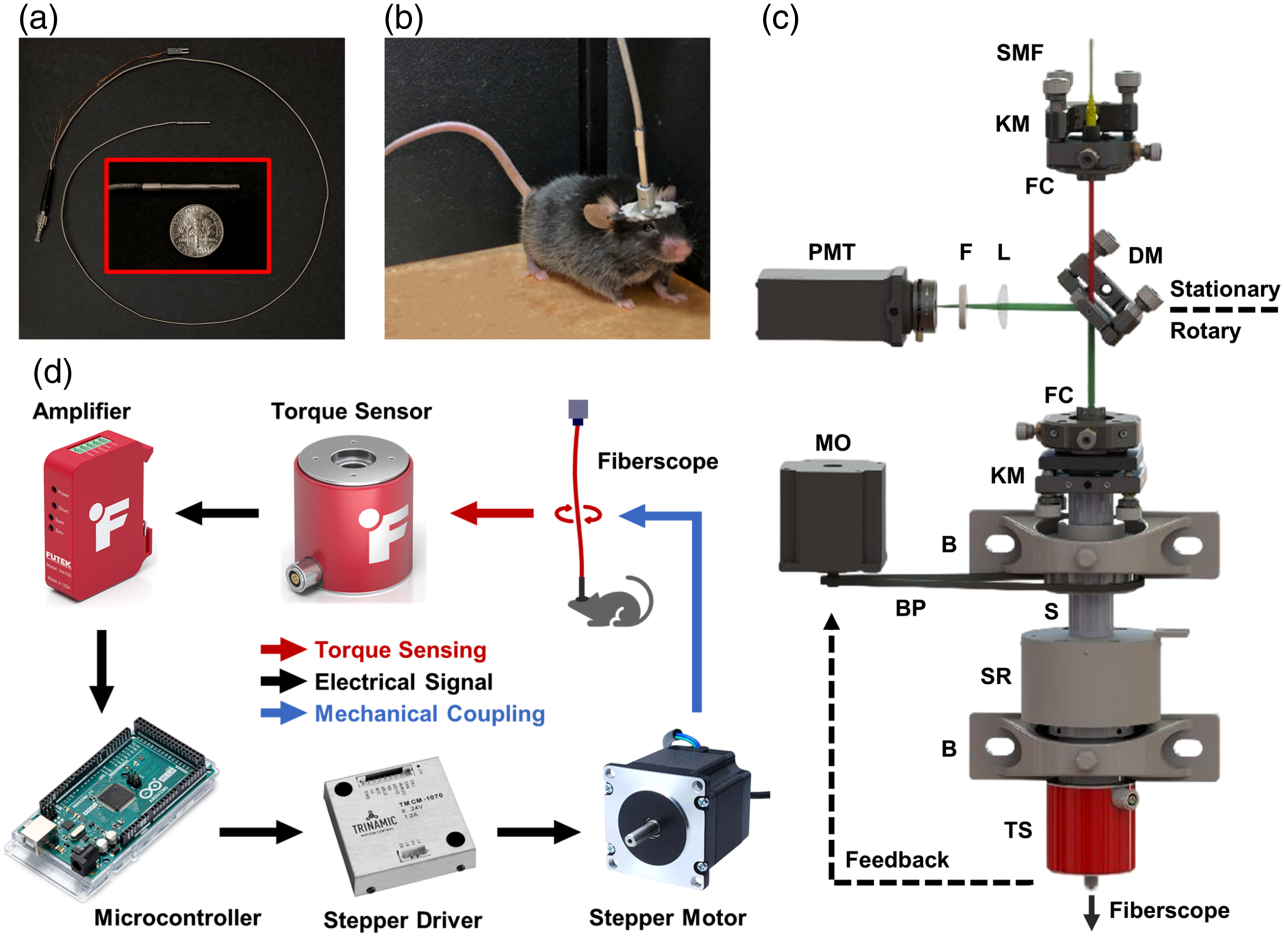
pOEC for 2P fiberscope neuroimaging in freely moving mouse. (a) Photo of an ultracompact fiberscope. Inset: the probe head next to a US dime. (b) Photo of a mouse with a head-mounted two-photon (2P) fiberscope. (c) Schematic of the pOEC system for 2P imaging. SMF, single-mode fiber; KM, kinematic mount; FC, fiber collimator; DM, dichroic mirror; L, lens; F, optical filter; PMT, photomultiplier tube; MO, stepper motor; B, bearing; S, shaft; BP, belt/pulley; SR, slip ring; TS, torque sensor. (d) Feedback control loop for proactive torque sensing and compensation.

**Fig. 2:**
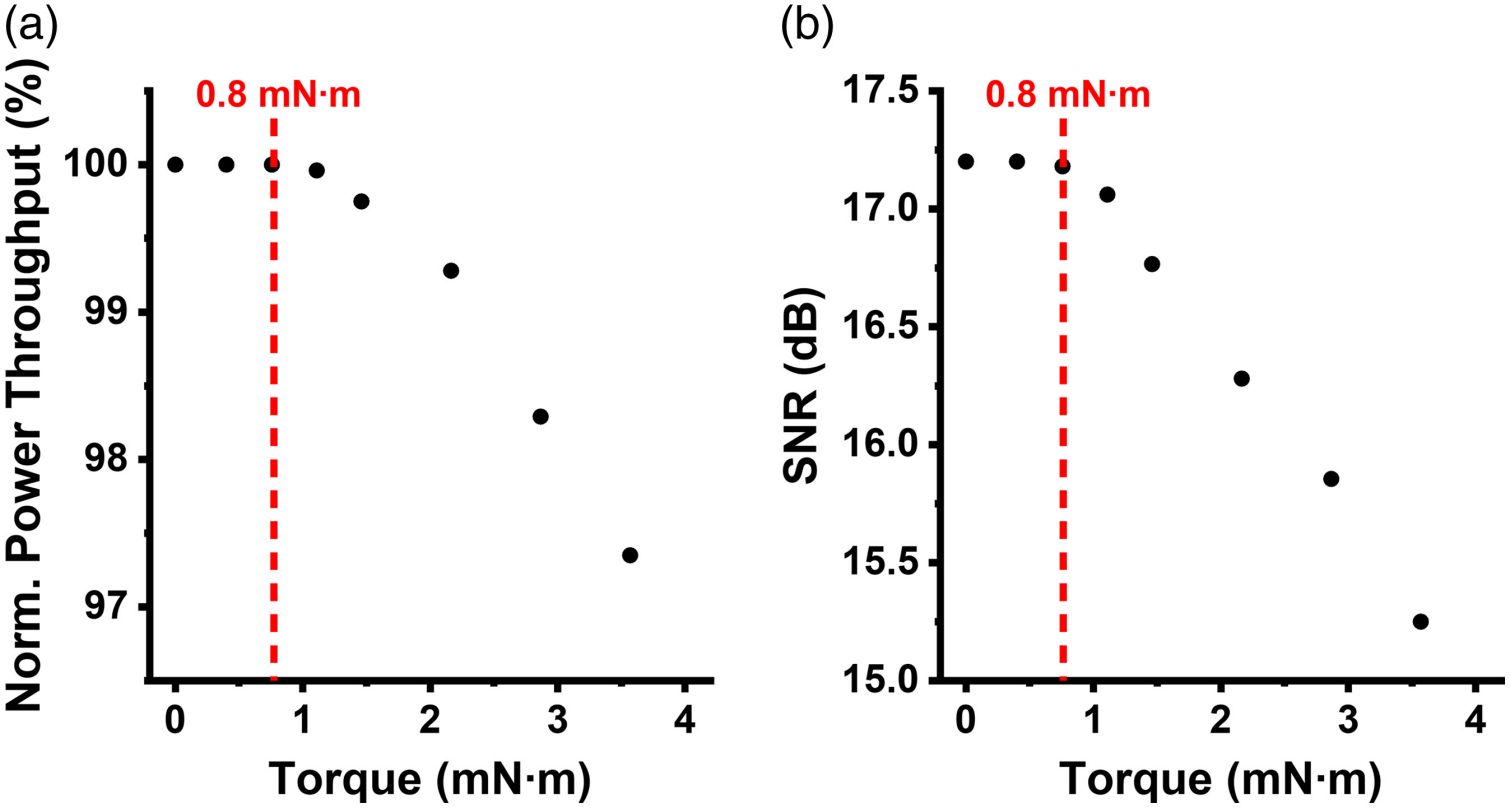
Impact of twisting on laser power throughput and SNR. (a) The measured normalized power throughput of the 2P fiberscope under varying twist conditions. No power loss was observed below the 0.8 mN·m threshold. (b) The measured SNR of the fiberscope versus the torque buildup in the tether. No decrease in SNR was observed below the 0.8 mN·m threshold.

The 2P fiberscope used in this work has a similar design to our previous work,^19^^,^^23^^,^^24^ with a free-hanging composite cantilever tip, a tubular piezoelectric actuator, and a home-built micro-objective, housed inside a hypodermic tube [housing outer diameter (OD): 2.8 mm, fiberscope weight: 1.3 g, see Fig. 1(a)]. The fiberscope provides a resolution of 1.2×15.9  μm at 920 nm and a FOV of over 500  μm in diameter. Figure 1(b) shows the photo of the 2P fiberscope mounted on the head of a freely behaving mouse for real-time neuroimaging (primary motor cortex). The proximal end of the fiberscope was connected to an optoelectrical commutator (OEC). An ideal OEC should maintain a highly stable optical and electrical coupling/connection between the nonmoving base instrument and the moving fiberscope over its 360 deg rotation. Drawing from our previous study on endoscopic optical coherence tomography^25^ and 2P fiberscope^23^ [Fig. 2(a)], a fiber-optic rotary joint can achieve a steady optical coupling. An excellent fiber core-to-core coupling efficiency with a less than ±1% coupling fluctuation over 360 deg rotation can be accomplished, and such a small fluctuation is negligible for 2P imaging of neuronal calcium dynamics. The electrical connection during rotation can be achieved by incorporating a commercially available hollow slip ring. Figure 1(c) illustrates the schematic of the pOEC, which includes a pair of fiber-optic collimators (FC) with one fixed on the stationary base platform and the other mounted on a rotary shaft. When the two FCs and the shaft are colinearly aligned, the excitation beam from the input single-mode fiber (SMF, shown in red) could be coupled into the proximal end of the 2P fiberscope (i.e., the single-mode core of the DCF) with minimum laser power throughput fluctuation during rotation. The fluorescence photons (shown in green) collected by the fiberscope travel through the outer cladding (and the core) of the DCF and are then collimated by the FC and reflected by a dichroic mirror (DM) to a photomultiplier tube (PMT) for detection. The electrical drive wires for the fiber-optic scanner at the distal end of the fiberscope pass through the core of the hollow shaft and are then connected to the base platform via a slip ring.

The crucial component of our pOEC is the torque sensor (FUTEK TFF400), with a sensitivity threshold sufficiently small (0.2 mN·m) to proactively sense any tiny torque build-up in the fiberscope. Its torque capacity is large enough to withstand the torques exerted by the animal. A real-time feedback control was developed to proactively compensate for the tiny torque (at a preset threshold) in the fiberscope when it is slightly twisted by the animal. The feedback loop operation is given in Fig. 1(d). When the torque sensor detects a tiny twist in the fiberscope (even before any perceptible rotation), it converts the torque to an electrical signal and sends it to a microcontroller (Arduino Mega 2560). The microcontroller constantly monitors the signal. Once the voltage of the signal passes a preset threshold, the microcontroller will drive the stepper motor (Jinwen 120027 with an angular resolution of 0.225 deg/microstep), which mechanically rotates the shaft to compensate for the torque in the fiberscope. Two critical preselected parameters determine the amount of rotational resistance the pOEC exerts on the animal: the torque threshold (the minimum torque required to activate the torque compensation mechanism) and the response time of the stepper driver. The threshold should be sufficiently small to ensure proactive torque sensing but not too small to trigger the compensation mechanism and induce unwanted small vibrations of the fiberscope. The response time of the stepper driver should be sufficiently fast to ensure real-time compensation but not too fast to risk overcompensating any self-oscillation of the system. To determine the appropriate parameters and optimize the overall performance of the pOEC, preliminary experiments have been conducted on mouse models (adult mice, 8 to 12 weeks old, 25 g or larger). In these experiments, we set the torque threshold at 0.8 mN·m and the corresponding stepper driver response time to 1.2  μs. To create such torque, an adult mouse only needs to exert around 1.5 g of force on its front or rear limbs, which is significantly below the 50 g force that an adult mouse can generate.^26^ In addition, with this low torque threshold and the excellent fiber core-to-core coupling efficiency (<±1% coupling fluctuation), the fiberscope does not experience observable loss in imaging quality over 360 deg rotation. The Arduino code for torque compensation is provided in the Supplemental Material.

The twisting of optical fibers can also induce fluctuations in laser power throughput. Previous studies have reported twist-induced power loss in SMF.^27^^,^^28^ For our fiberscope that is fabricated using a customized DCF, we measured the one-way power throughput of the fiberscope under varying twist conditions, and the results are presented in Fig. 2(a). No loss in power throughput was observed at 0.8 mN·m, the threshold for triggering the pOEC torque compensation mechanism. Furthermore, we investigated the effect of twisting on 2P imaging quality, taking into account its impact on both the excitation light power throughput and the collection of emission light. We measured the relationship between the signal-to-noise ratio (SNR) of the fiberscope and the torque buildup in the tether. Torque in the tether was measured using the torque sensor, and fluorescence photons were recorded using a PMT (emission filter: 525±10  nm) while imaging a green fluorescent reference slide under femtosecond laser excitation (920 nm, 3 mW). PMT signals recorded with laser excitation were treated as signals, whereas those recorded without excitation were considered noise. The results in Fig. 2(b) demonstrated that the SNR remained unchanged when the torque buildup in the tether was below the 0.8 mN·m threshold.

### 2.2 Animal Model

#### 2.2.1 Animal model preparation

In this work, we used nine male Camk2-Cre mice (Jax, #005359). Six mice were employed to investigate their motor behavior and demonstrate the performance of the pOEC system, whereas the three other mice, expressing GCaMP6m in the primary motor cortex, were used for 2P neuroimaging and studying the impact of rotational resistance on their neural activity.

To obtain 2P neuroimages from the mice, cranial window surgeries were performed when the mice were 8 weeks old. During surgeries, the mice were anesthetized by inhalation of 2% isoflurane and $O_{2}$ and then locked to a stereotaxic platform. After removing the scalp and exposing the skull, a 4 mm diameter round craniotomy was drilled over the primary motor cortex. GCaMP6m virus (AAV/DJ-flex-GCaMP6m, Neuroconnectivity Core, Baylor College of Medicine) was injected into the target region via a glass microneedle (World Precision Instrument TIP10LT, 1mm O.D., 10  μm tip diameter) attached to a microinjector pump (Nanoject II, Drummond). After the injection, a #1 glass coverslip was placed on the exposed brain, and it was sealed to the skull with tissue adhesive (3M Vetbond). A customized titanium-restraining bar was glued to the head with dental cement for attaching the endomicroscope later. The transgene expression was checked 3 to 4 weeks post surgeries with a tabletop 2P microscope.

#### 2.2.2 Ethics statement

All animal experiments comply with the ARRIVE guidelines and were carried out in accordance with the National Institutes of Health Guide for the Care and Use of Laboratory Animals. All data reported in this study were collected at Johns Hopkins University with the approval from JHU Animal Care and Use Committee.

### 2.3 Neural Data Processing

The GCaMP fluorescence neural data were analyzed using our post-processing pipeline developed in MATLAB and Python. First, we applied the NoRMCorre algorithm^29^ to correct motion artifacts in all images acquired by the 2P fiberscope and used the CANDLE filter^30^ for denoising. Then, we used a well-established pipeline CaImAn^31^ for the segmentation of neurons and the extraction of their temporal calcium dynamics (ΔF/F traces). Quantification of each neuron’s calcium activity (i.e., firing activities) was based on OASIS^32^ and published calcium activity analysis methods.^33^^,^^34^ Finally, we analyzed mice’s neural activity across different experimental conditions using our customized Python codes.

## 3 Results

### 3.1 Motion Behavior

The performance of the pOEC was evaluated by analyzing the movement of the mice with a head-mounted 2P fiberscope. The fiberscope had a 2.8 mm diameter at its distal end, and the details about the fiberscope were reported elsewhere.^22^^,^^23^ Three different experimentation conditions were used to assess the performance of the pOEC: (1) Free: in this condition, the proximal end of the fiberscope was connected to a very small bearing [inner diameter (ID): 2.9 mm, OD: 6.0 mm] capable of smooth rotation with negligible rotational resistance and inertia. (2) On: the fiberscope was connected to the pOEC with torque compensation enabled. (3) Off: it is similar to (2) but with the torque compensation disabled. It is important to emphasize that the first condition aimed to simulate a scenario of complete movement freedom for the mouse except for having a fiberscope mounted on its head. Although untethered mice have the capability for arbitrary movement, our observation indicated that they were less active and tended to seek more rest (see results in the Appendix). Therefore, it is more appropriate to compare the motion behaviors among tethered mice.

**Fig. 3:**
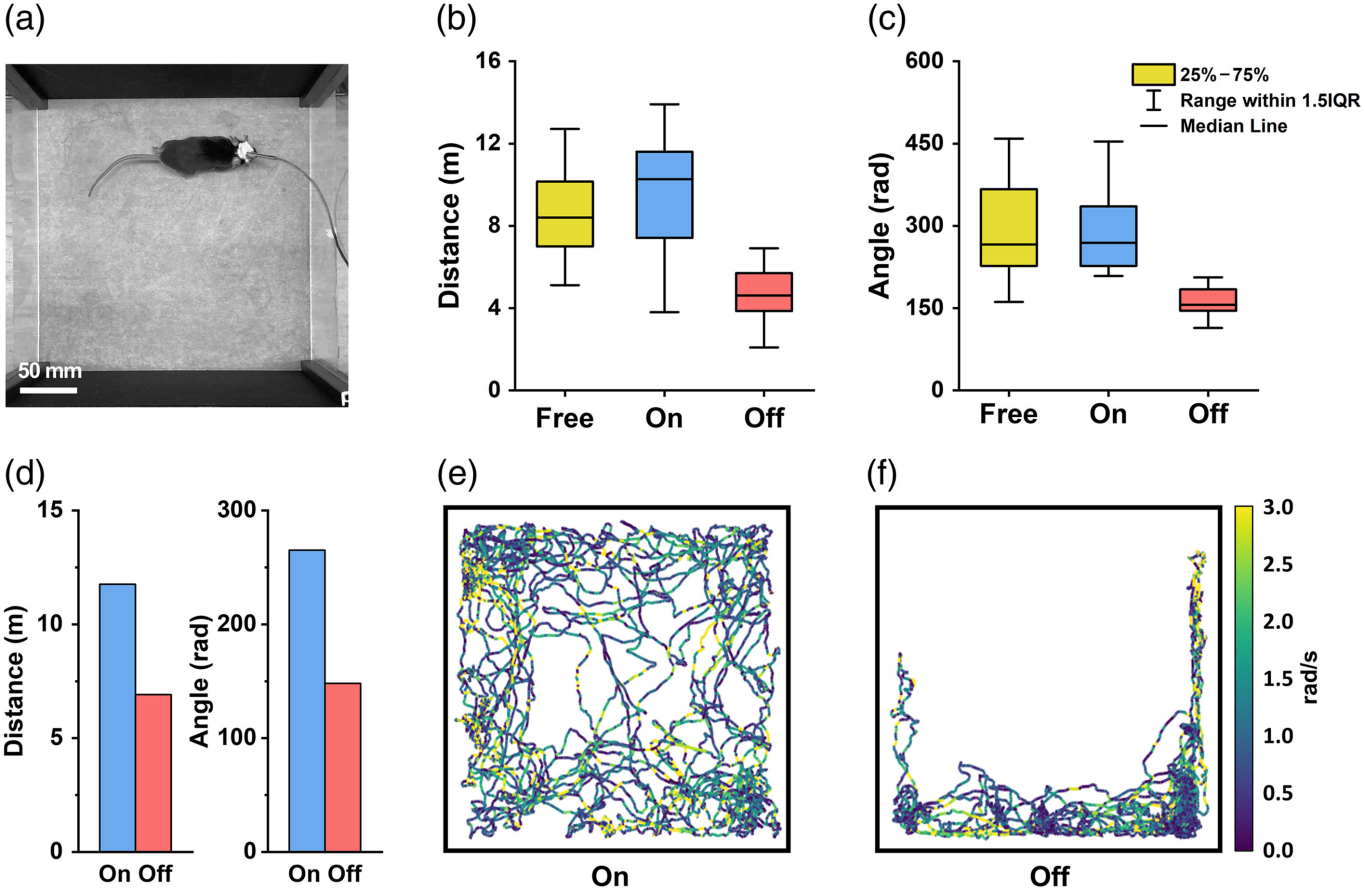
Comparison of the movements of mice with a head-mounted fiberscope under three conditions of the pOEC: (1) Free, marked in yellow. (2) On, marked in blue. (3) Off, marked in red. (a) Photo of a representative mouse walking in a square arena wearing a fiberscope. (b) Box-and-whisker plot of the distance a mouse traveled within one session. (c) Box-and-whisker plot of the angle a mouse turned within one session. Sample: six mice; center line, median; limits, 75% and 25%; whiskers, maximum and minimum. (d) Representative mouse movement under “on” and “off” conditions. Left: total walking distance in one imaging session. Right: total turning angle in the session. (e) Movement trajectory corresponding to the “on” condition in panel (d). (f) Movement trajectory corresponding to the “off” condition in panel (d). Trace color indicates the angular speed of the mouse (unit: rad/s). Mouse’s movement recording with the pOEC function turned on (see Video 1, MP4, 9.46 MB [URL: https://doi.org/10.1117/1.NPh.12.2.025016.s1]).

Throughout the experiment, each of the six mice was released into a square open arena [$10^{\prime \prime }\times 10^{\prime \prime }$ in size, see Fig. 3(a)] for three 333-s sessions under each condition (free, on, and off), with intervals of more than 5 min between two adjacent sessions to ensure sufficient rest for the mouse. The mice’s movements were video recorded, and the movement trajectories were extracted by utilizing DeepLabCut,^35^ a state-of-the-art markerless pose estimation toolbox. The analyses of mice’s walking and turning movements were conducted in Python using our customized codes. To evaluate the mice’s freedom of movement, we calculated their walking distance, turning angle, as well as their angular speed during turning. Box-and-whisker plots of the walking distance and turning angle of the six mice (18 measurements under each condition) were shown in Figs. 3(b) and 3(c), respectively, revealing that mice were more active when proactive torque compensation was enabled compared with when it was disabled. Representative movement trajectories of one mouse under “on” and “off” conditions were extracted and displayed in Figs. 3(e) and 3(f), with the corresponding walking distance and turning angle plotted in Fig. 3(d). In the absence of torque compensation, the mouse’s trajectory remained confined to a specific area with a relatively lower angular speed, rather than freely roaming over the entire arena.

Statistical analysis was conducted in SPSS, with a significance level set at 0.05. As our data involved repeated measurements of the same six mice under three experimental conditions, we employed a mixed-effects model to assess the impact of the three conditions on mice’s movement. In the model, the three conditions were treated as a fixed effect, whereas the individual differences between mice and the variations between measurements were accounted for as nested random effects. Significant differences were observed in both walking distance (p=0.0056) and turning angle (p=0.0090) across three conditions. To further analyze differences between specific conditions, Bonferroni post hoc tests were performed, and the resulting p-values are presented in Tables 1 and 2. The tests revealed significant differences between the “on” and “off” conditions in both walking distance (p=0.0008) and turning angle (p=0.0232). When torque compensation was “on,” the mice’s traversed distance per session (mean: 9.51 m) increased by an average of 103% compared with the “off” state (mean: 4.67 m). Similarly, the total angle turned per session increased by an average of 79% with torque compensation enabled (mean: 287.15 rad), compared with the disabled condition (mean: 160.20 rad). However, no significant differences were observed between the “on” and “free” conditions in terms of both walking distance (p=1) and turning angle (p=1), suggesting that the mice experienced a similar level of movement freedom when torque compensation was enable compared with the freely rotating condition. In fact, the mean values of walking distance and turning angle under these two conditions were very close, indicating that our pOEC-based system generated nearly zero rotational resistance to the animals.

**Table 1:** Mean values of mice’s total walking distance under three conditions and p-values from Bonferroni post hoc tests.

Condition	Mean (m)	Comparison	p-value
Free	8.60	On versus Off	0.0008
On	9.51	Free versus Off	0.0104
Off	4.67	On versus Free	1

**Table 2:** Mean values of mice’s total turning angle under three conditions and p-values from Bonferroni post hoc tests.

Condition	Mean (rad)	Comparison	p-value
Free	288.29	On versus Off	0.0232
On	287.15	Free versus Off	0.0239
Off	160.20	On versus Free	1

### 3.2 Neural activity

**Fig. 4:**
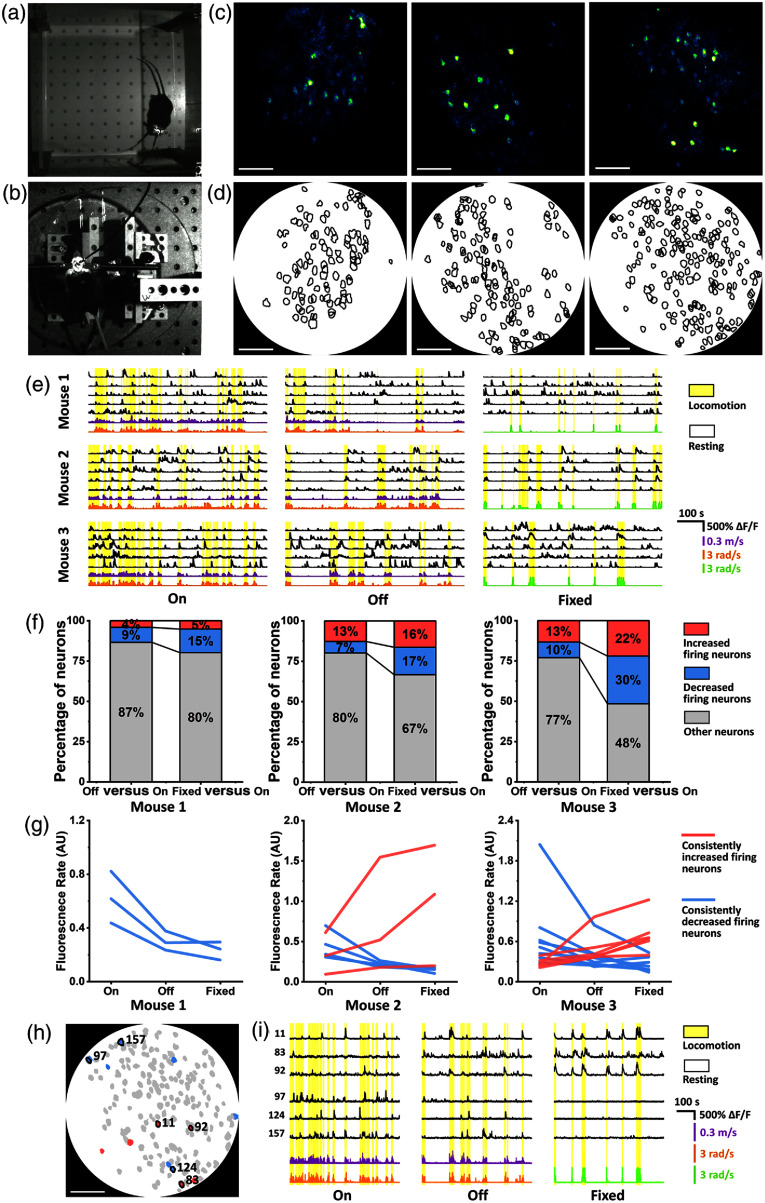
Neuroimaging in freely behaving and head-fixed mice and the influence of rotational resistance on neural activity. (a) Photo of a freely behaving mouse in a square arena wearing a fiberscope. (b) Photo of a head-fixed mouse on a disk-shaped platter with a head-mounted fiberscope. (c) Representative GCaMP6m 2P images of mice’s firing motor cortex neurons. All images were acquired at a frame rate of 3 fps without frame averaging. From left to right: mouse 1, mouse 2, and mouse 3. FOV: 500  μm in diameter. Scale bars: 100  μm. (d) Segmentation masks of the identified neurons. From left to right: mouse 1 (101 neurons), mouse 2 (143 neurons), and mouse 3 (198 neurons). Scale bars: 100  μm. (e) Representative ΔF/F curves of five neurons for each mouse over the total 6000 imaging frames along with the mouse’s linear and angular velocity trace (marked in purple and orange, respectively) under the “on” and “off” conditions, and the platter’s rotational angular speed trace (marked in green) under the “fixed” condition. (f) Stacked percentage bar plot of three categories of neurons for three mice. The first column in each subplot shows the following: comparing “off” to “on” conditions, neurons with significantly increased firing activities are marked in red, neurons with significantly decreased firing activities are marked in blue, and the other neurons with minor overall firing changes are marked in gray. The second column in each subplot shows the proportions of three types of neurons when comparing “fixed” to “on” conditions. (g) Median fluorescence rates of each mouse’s neurons under three different conditions. Neurons with consistently increased fluorescence rates (i.e., $R_{\text{off}}>R_{\text{on}}$ and $R_{\text{fix}}>R_{\text{on}}$) are marked in red, whereas those with consistently decreased fluorescence rates (i.e., $R_{\text{off}}<R_{\text{on}}$ and $R_{\text{fix}}<R_{\text{on}}$) are marked in blue. (h) Segmentation mask of mouse 3’s neurons with colors indicating the categories is shown in panel (g). (i) ΔF/F curves of six neurons highlighted in panel (h), along with mouse 3’s linear and angular velocity traces (purple and orange) and the platter’s angular velocity trace (green).

To investigate the influence of rotational resistance from the tether on mice’s neural activity, we performed 2P neuroimaging on freely behaving mice under two conditions: (1) On: a mouse was set free in the square arena [see Fig. 4(a)], with torque compensation of the pOEC system enabled. (2) Off: torque compensation was disabled. In addition, as brain imaging on head-fixed animals has been a commonly used model in neuroscience research, we also explored the potential impact of head restraint on neural activity. Thus, we performed 2P neuroimaging under another condition. (3) Fixed: the mouse was placed on a disk-shaped platter, which can rotate smoothly with minimal resistance but with its head fixed [see Fig. 4(b)]. Its head was fixed with a head-restraining bar to a frame above the platter, whereas its body was free to move. Here, we treated the “on” condition as a baseline, where the mouse was freely moving/rotating with almost zero rotational resistance. Under the “off” condition, the mouse had to overcome the rotational resistance from the tether when moving and could not rotate freely. Under the “fixed,” the mouse could not move its head at all, and the rotational resistance was invincible.

Three mice with GCaMP6 expression in the primary motor cortex were used in this study. During the experiment, each mouse was imaged for two 1000-frame (333 s) imaging sessions under each condition, with intervals of more than 5 min to allow the mouse to rest. Both 2P fluorescence neuron images and movements of the mouse were recorded simultaneously. Representative 2P calcium images of the three mice’s motor cortex neurons (pOEC function, on; FOV, 500  μm) were shown in Fig. 4(c). With a total of 6000 imaging frames for each mouse, individual neurons were identified, and their segmentation masks were plotted in Fig. 4(d). The ΔF/F traces of each neuron were extracted over all imaging sessions. Movement trajectories of each mouse during freely behaving sessions were extracted from the video recordings, and their moving velocities were calculated. To distinguish locomotion and resting periods in freely behaving mice, we applied a velocity threshold. A value of 1  cm/s was chosen, considering both the mice’s activity levels and the size of the behavioral arena.^36^ The periods identified using this threshold closely matched the mice’s movement patterns observed in the video recordings. In head-fixed sessions, where the mouse walked on a rotating platter, the platter’s angular rotation was used to infer locomotion. We calculated the rotational angular velocity of the platter and applied a threshold equivalent to 1  cm/s of linear movement to define locomotion and resting periods for the head-fixed condition. The calcium dynamics of five representative neurons from each mouse are shown in Fig. 4(e), along with the mouse’s movement velocity during freely behaving sessions and the platter’s rotational angular velocity during head-fixed sessions, with locomotion periods highlighted in yellow.

To analyze neural activity quantitatively, we calculated the fluorescence rate of neuronal firing during locomotion periods for all identified neurons under three experimental conditions. For each neuron, the fluorescence rate of an individual firing was defined as the area under a firing peak within the ΔF/F trace divided by firing duration time, i.e., r=ΔS/Δt. Under each condition, the neuron’s fluorescence rates of a given neuron were represented as sets of values: $R_{\text{on}}=\{r_{1},r_{2},\ldots ,r_{i}\}$, $R_{\text{off}}=\{r_{1},r_{2},\ldots ,r_{j}\}$, and $R_{\text{fixed}}=\{r_{1},r_{2},\ldots ,r_{k}\}$, where i, j, and k denote the numbers of firing events observed in each condition. These numbers vary depending on the activity level of the neuron. Normality tests revealed that the distributions of fluorescence rates did not follow a normal distribution. Thus, we employed the Mann–Whitney U test for statistical comparisons of neuronal activity across conditions, with a significance level set at 0.05.

First, we compared the neural activity between the “off” and “on” conditions (Off versus On). Neurons, the overall fluorescence rate of which in the “off” condition was significantly higher than in the “on” condition ($R_{\text{off}}>R_{\text{on}}$, p<0.05), were classified as increased firing neurons. Conversely, neurons exhibiting a significantly lower overall fluorescence rate in the “off” condition than in the “on” condition ($R_{\text{off}}<R_{\text{on}}$, p<0.05) were classified as decreased firing neurons. The remaining neurons that did not show a statistically significant difference in fluorescence rate between the two conditions are categorized as other neurons. Then, we performed a similar comparison between the “fixed” and “on” conditions (Fixed versus On). Based on the same criteria, neurons were classified as increased firing neurons ($R_{\text{fixed}}>R_{\text{on}}$, p<0.05), decreased firing neurons ($R_{\text{fixed}}>R_{\text{on}}$, p<0.05), and other neurons. The percentage of each neuron category relative to the total number of neurons is shown in Fig. 4(f). Taking mouse 1 as an example, with the “on” condition as the baseline, switching to the “off” condition resulted in 4% of neurons increasing their firing activity, 9% decreasing their firing activity, and 87% exhibiting no obvious differences. When switching to the “fixed” condition, 5% of neurons showed increased firing, 15% showed decreased firing, and the percentage of neurons with no significant changes dropped to 80%. Based on data from the three mice, overall, as we compared the condition transitions from “on” to “off” and then to “fixed,” the proportion of neurons exhibiting statistical changes in fluorescence rates increased progressively (mouse 1: 13% → 20%; mouse 2: 20% → 33%; and mouse 3: 23% → 52%). Specifically, in all three mice, the proportion of neurons with increased firing activity rose slightly, whereas the proportion of neurons with decreased firing activity rose more substantially (e.g., mouse 3: 10% → 30%).

In addition, we examined neurons that consistently exhibited increased firing activity in both the “off” and “fixed” conditions compared with the “on” condition (i.e., $R_{\text{off}}>R_{\text{on}}$ and $R_{\text{fix}}>R_{\text{on}}$) Similarly, we identified neurons that consistently showed decreased firing activity in both conditions (i.e., $R_{\text{off}}<R_{\text{on}}$ and $R_{\text{fix}}<R_{\text{on}}$). The median fluorescence rates of these neurons under three conditions are highlighted in Fig. 4(g). We observed that the majority of the neurons—except for one neuron in mouse 1 and one in mouse 3—showed a continuous increasing or decreasing trend in their median fluorescence rates with increasing rotational resistance, from the “on” condition to the “off” and then to the “fixed” condition.

Furthermore, we investigated the neurons in mouse 3 that exhibited consistently increased or decreased firing and marked them in the segmentation masks, as shown in Fig. 4(h). Three representative neurons of each type were highlighted, and their ΔF/F traces were displayed in Fig. 4(i), illustrating detailed calcium transients during locomotion periods under different conditions. Neurons 11, 83, and 92 exhibited more frequent firing events and higher ΔF/F peaks in the “off” and “fixed” conditions compared with the “on” condition. By contrast, neurons 97, 124, and 157 were significantly more active in the “on” condition, exhibiting a greater number of firing events.

## 4 Discussion and Conclusion

In this study, we developed a pOEC by integrating a sensitive torque sensor for sensing the twist/torque buildup in the head-mounted fiberscope, and a feedback loop for proactive torque compensation in real time. The pOEC can relieve the animal from heavy restraining force and prevent the fiberscope tethered to the animal’s head from twist-breaking. The implementation of proactive torque compensation has shown a significant improvement in the animal’s freedom of movement.

Our previous work^23^ demonstrated an OEC that used a rotary encoder to measure the twist angle of the fiberscope tether and a motor to reactively compensate for the rotational displacement. That system required a minimum angular displacement of 2 deg, equivalent to a torque of 8  mN·m (roughly measured by a pulley and weight setup), to activate the compensation mechanism. In comparison, our pOEC system precisely measures the torque buildup in the tether and proactively compensates for the torque with a much lower threshold of 0.8  mN·m. This means a force 10 times lower—∼1.5  g, generated by a mouse’s front or rear limbs—can activate the pOEC compensation mechanism, making the resistance from the tether almost imperceptible to the mouse.

In our 2P fiberscope, both excitation light delivery and fluorescence signal collection go through the same DCF. We investigated the effect of twisting the optical fiber on laser power throughput. No drop in throughput was detected when the torque buildup in the tether of the fiberscope was below 0.8  mN·m threshold. We further evaluated the impact of fiber twisting on 2P imaging quality, considering the double pass of excitation and emission light; no degradation in SNR was observed the torque remained below 0.8  mN·m. These results demonstrate the effectiveness of the pOEC system in maintaining optical performance under negligible rotational resistance.

2P neuroimaging in freely rotating mice was achieved using an ultracompact 2P fiberscope along with the pOEC system. The torque compensation method allowed investigation of the impact of rotational resistance caused by the head-mounted tether on mouse neural activity. In addition, we examined an extreme condition—head-fixed, where the mouse’s head experienced great resistance and was completely immobilized—to assess its effect on neural activity. Analyses of 2P calcium images revealed that, in all three tested mice, a subset of neurons exhibited statistically significant increases or decreases in their fluorescence rates under the “off” condition compared with the “on” condition (mouse 1: 13%, mouse 2: 20%, and mouse 3: 23%). Under the “fixed” condition, an even larger proportion of neurons showed significant changes in fluorescence activity (mouse 1: 20%, mouse 2: 33%, and mouse 3: 52%). The results indicate a trend that a considerable portion of neurons is affected when the mouse is under an experimental condition (restricted by the rotational resistance from a tether or completely unable to turn due to head fixation) that deviates further from its natural physiological state (freely moving/rotating). The presence of rotational resistance appears to influence the overall activity level of a nonnegligible population of neurons, potentially confounding the interpretation of neural dynamics and behavior. Therefore, performing neural imaging in freely moving and rotating animals is preferable for accurately studying neural function.

In addition, we observed some neurons, the fluorescence rates of which consistently increased or decreased as the rotational resistance on the mouse was higher from the “on” condition to the “off” and then to the “fixed” condition. These neurons might be rotation-sensitive, and their neural activity could be more susceptible to rotational resistance. Although the specific role of certain motor cortex neurons or areas in walking/rotating behaviors requires further investigation, our pOEC system holds the potential to provide valuable information on the function of the motor cortex and offers profound scientific insight into population coding by motor cortex associated with animals’ motion behavior.

The unique advantage of the pOEC fiberscope system, allowing animals to behave freely under nearly natural conditions, positions itself as a potentially powerful tool for investigating motor cortex function through real (versus virtual^5^^,^^37^^–^^39^) exploration or navigation experiments. This advantage can extend to studies in various domains such as social behavior, memory, and cognition, which could benefit from allowing animals to move freely and observing their natural head, neck, and body movements. More importantly, our design is not limited to neuroimaging of rodents; it can potentially be applied to nonhuman primates such as rhesus macaque and marmoset. The capability to accommodate arbitrary rotations of animals would be particularly valuable when using heavier rotary imaging devices.

## 5 Appendix

**Fig. 5:**
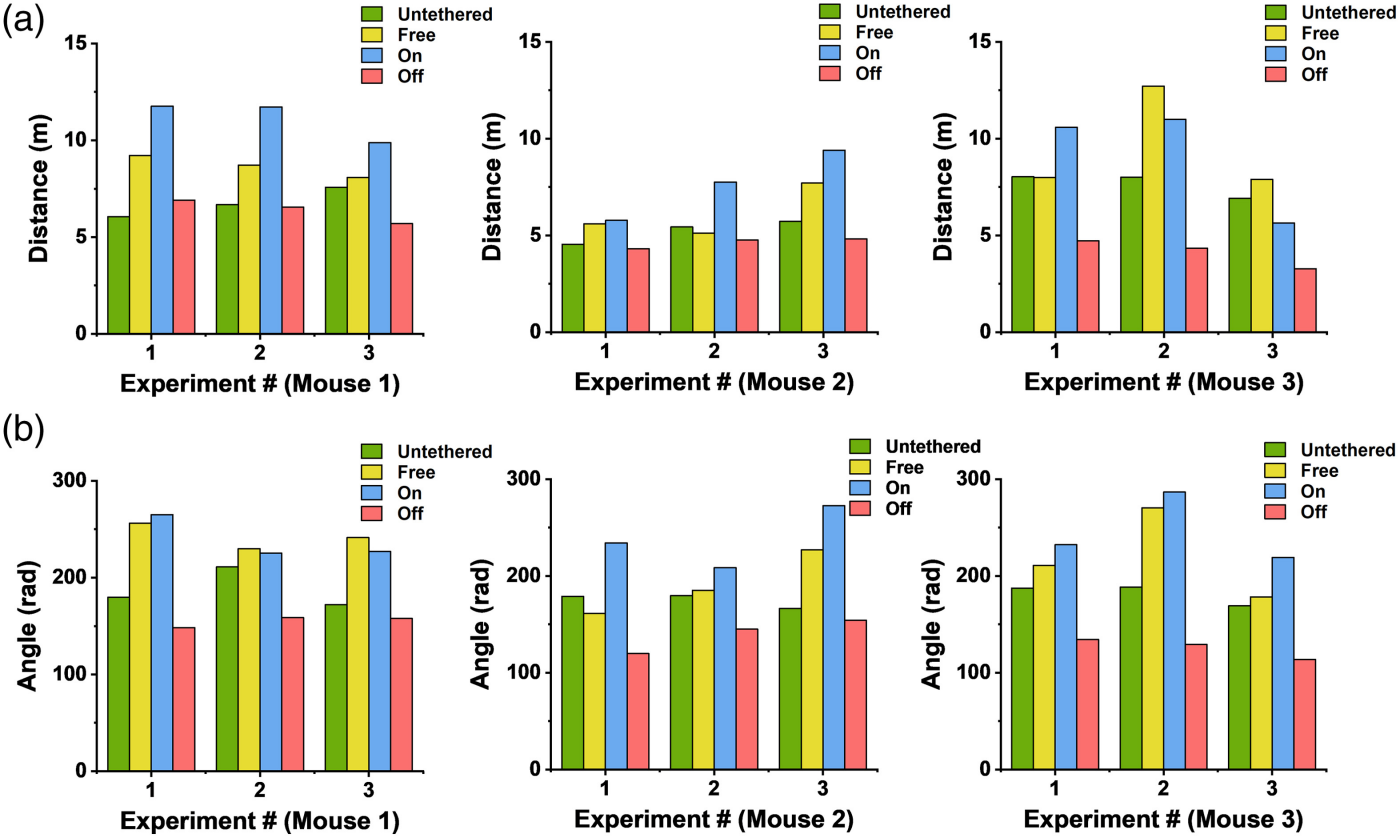
Behavior of three mice under untethered or tethered (Free/On/Off) conditions. Three experiments with each containing three sessions in different conditions were recorded for each mouse (mouse 1, mouse 2, and mouse 3). The bar charts show (a) the calculated walking distance and (b) turning angle of each mouse in three experimental sessions. From left to right: mouse 1, mouse 2, and mouse 3. Untethered (green color), Free: fiberscope connected to a small smooth bearing (yellow color), pOEC functionality on (blue color) and off (red color).

The behavioral results of three mice under untethered and tethered conditions are presented in Fig. 5.

## Supplementary Material

10.1117/1.NPh.12.2.025016.s01

10.1117/1.NPh.12.2.025016.s1

## Data Availability

Data will be made available on request.
