# Supplementary material for: Two-photon fiberscope with a proactive optoelectrical commutator for rotational resistance–free imaging in freely behaving rodents
Source: Neurophotonics. 2025 Jun 18;12(2):025016. doi: 10.1117/1.NPh.12.2.025016 (PMC12175966; doi:10.1117/1.NPh.12.2.025016)
Supplement: Supplementary file 1 [file NPh_012_025016_SD001.pdf]

Two-photon fiberscope with a proactive optoelectrical commutator for rotational resistance-free imaging in freely-behaving rodents

Supplementary Arduino code

```
#include <EEPROM.h>
#define CTRL_LOOP 1

enum MODE {FB, JOG};
MODE mode = JOG;
int pwmPin = 2;
int adcPin = 0;
int dirPin = 23;
int enaPin = 24;
int pulseRemain = 0;
int current = 0;
int target = 0;
int dir = 0;
String inString = "";
String cmd = "";
int forceSensor_pin = A8;
int forceCnt=0;
int force_bias = 511; // bias
float force_scale = 1.0; // gain
float curForce = 0.0;
float forceRange = 10.0; // threshold

int ledPin = 53;
int ledSuppPin = 52;
int monitorPin = 40;
int loopCnt = 0;


void pulse(int pin) {
  digitalWrite(pin, HIGH);
  delayMicroseconds(1200);
  digitalWrite(pin, LOW);
}

void setup() {

  // put your setup code here, to run once:
  pinMode(pwmPin, OUTPUT);
  pinMode(dirPin, OUTPUT);
```

```
  pinMode(enaPin, OUTPUT);
  pinMode(ledPin, OUTPUT);
  pinMode(ledSuppPin, INPUT);
  pinMode(monitorPin, OUTPUT);
  digitalWrite(dirPin, HIGH);
  digitalWrite(enaPin, HIGH);
  Serial.begin(115200); // baud
  while (!Serial) {
    ;
  }

  noInterrupts();           // disable all interrupts
  TCCR1A = 0;
  TCCR1B = 0;
  TCNT1  = 0;

  OCR1A = 32;
  TCCR1B |= (1 << WGM12);
  TCCR1B |= (1 << CS12);
  TIMSK1 |= (1 << OCIE1A);
  interrupts();             // enable all interrupts
  current = (EEPROM.read(0) << 8) | EEPROM.read(1);
  target = current;

}

ISR(TIMER1_COMPA_vect)
{
  digitalWrite(monitorPin, HIGH);
  switch (mode) {
    case FB:                    // Feedback tracking
      digitalWrite(ledPin, HIGH);
      if (curForce > forceRange && curForce < 200) //Offset 512
      {
        digitalWrite(dirPin, LOW);
        current--;
        pulse(pwmPin);
      }
      if (curForce < -forceRange && curForce > -200)
      {
        digitalWrite(dirPin, HIGH);
        current++;
        pulse(pwmPin);
      }
      break;
```

```
    case JOG:               // Jogging
      digitalWrite(ledPin, LOW);
      if (current != target)
      {
        if (current < target) {
          digitalWrite(dirPin, HIGH);
          current++;
        }
        if (current > target) {
          digitalWrite(dirPin, LOW);
          current--;
        }
        pulse(pwmPin);

      }
      break;
  }
  if (loopCnt == CTRL_LOOP)
  {
    loopCnt = 0;
    forceCnt = analogRead(forceSensor_pin);
    curForce = float(force_bias - forceCnt)*force_scale;
    Serial.println(curForce);
    //Serial.print('\n');
  } else loopCnt++;
  digitalWrite(monitorPin, LOW);
}

void loop() {
  while (Serial.available() > 0) {
    int inChar = Serial.read();
    if (isAscii(inChar)) inString += (char)inChar;
    if (inChar == '\n') {
      cmd = inString + '\0';
      inString = "";
      if (cmd[0] == 'J') //Jogging
      {
        String jogCount = cmd.substring(1);
        if (jogCount.length() > 0) target = current + jogCount.toInt();
        else target = current;
        mode = JOG;
        cmd = "";
      }
      if (cmd[0] == 'F') //Tracking
      {
```

```
        //enc.write(0);
        mode = FB;
        cmd = "";
      }
      if (cmd[0] == 'R') //Reset
      {
        EEPROM.write(0, 0);
        EEPROM.write(1, 0);
        EEPROM.write(2, 0);
        EEPROM.write(3, 0);
        current = 0;
        target = 0;
        cmd = "";
      }
    }
  }
}
```
